# The multi-targeted tyrosine kinase inhibitor vandetanib plays a bifunctional role in non-small cell lung cancer cells

**DOI:** 10.1038/srep08629

**Published:** 2015-02-27

**Authors:** Yan Zhou, Yuanliang Zhang, Hanbing Zou, Ning Cai, Xiaojing Chen, Longmei Xu, Xianming Kong, Peifeng Liu

**Affiliations:** 1 Central Laboratory, Ren Ji Hospital, School of Medicine, Shanghai Jiao Tong University, Shanghai. 200127, People's Republic of China; 2 Shanghai Institute of Hematology, Ruijin Hospital, School of Medicine, Shanghai Jiaotong University, Shanghai. 200025, People's Republic of China; 3 State Key Laboratory of Oncogenes and Related Genes, Shanghai Cancer Institute, Ren Ji Hospital, School of Medicine, Shanghai Jiao Tong University, Shanghai. 200032, People's Republic of China

## Abstract

Vandetanib, a multikinase inhibitor, is a target of drug treatments for non-small cell lung cancer (NSCLC). However, phase II and III clinical trials have not conclusively demonstrated the curative effects of vandetanib for NSCLC, and the reasons for this are unknown. In the present study, we use the NSCLC cell line Calu-6 as a model to determine the cellular and biological effects of vandetanib. Our results demonstrate that vandetanib impairs Calu-6 cell migration and invasion. We find that vandetanib can directly inhibit RET activity, which influences the Rho-JNK pathway. Overexpression of a constitutively active Rho GTPase antagonizes the inhibitory effects of vandetanib on Calu-6 cells invasion and JNK pathway activation. In addition, vandetanib induces autophagy by increasing the level of reactive oxygen species (ROS) in Calu-6 cells, and blockade of autophagy or ROS effectively enhances the cell death effect of vandetanib. In this study, we find vandetanib is of a double effect in some NSCLC cells, presenting new possibilities for the pharmacological treatment of NSCLC and introducing a novel role for vandetanib in treatment options.

Lung cancer is one of the most common cancers and non-small cell lung cancer (NSCLC) accounts for 80–85% of all lung cancers. Although effective treatments such as surgery, chemotherapy, and radiotherapy have been greatly improved, the 5-year survival rate for patients is still very low^1^, and there is an urgent need for better treatment options.

An epidermal growth factor receptor (EGFR) inhibitor has recently been developed and has been shown to be effective against NSCLC^2^ as more than 60% of NSCLCs express EGFR with genetic mutations. However, the emergence of drug-resistant variants of NSCLC has greatly reduced the clinical efficacy of EGFR inhibitors such as gefitinib^3^,^4^,^5^. Multiple tyrosine kinase inhibitors (TKIs), such as sorafenib, lapatinib, and vandetanib, have therefore been designed based on these drug-resistant variants^6^,^7^,^8^. Vandetanib acts as a TKI of cell receptors including EGFR, vascular endothelial growth factor receptor (VEGFR) and RET-tyrosine kinase^9^,^10^,^11^. The Food and Drug Administration (FDA) has approved vandetanib for the treatment of symptomatic or progressive medullary thyroid cancer in patients with unresectable locally advanced or metastatic disease. As mentioned above, EGFR is often mutated in lung cancer cells. In addition, VEGFR is required for tumor angiogenesis^12^, and KIF5B-RET translocation occurs in approximately 1–2% of lung adenocarcinoma^13^. These data indicate that vandetanib may represent a potential treatment option for NSCLC^14^,^15^. In initial studies, favorable outcomes for NSCLC patients (Progression Free Survival only) were observed in a phase II study evaluating vandetanib plus standard platinum-based front-line chemotherapy (007 trial) versus chemotherapy alone and in a phase III trial (ZODIAC) evaluating the addition of vandetanib to the standard second-line drug docetaxel. However, numerous phase II and III trials have failed to show any meaningful differences in terms of outcomes with the additional use of vandetanib for the treatment of NSCLC. Based on the negative results of phase III trials (ZEAL and ZEST), further evaluation of vandetanib as monotherapy or in combination with standard chemotherapies in unselected patients with NSCLC will be difficult. Hence, it is necessary to identify clinical and molecular biomarkers of patients who would benefit from vandetanib and, furthermore, to attempt to determine the molecular mechanism of drug resistance in patients.

Autophagy is a conserved pathway that is crucial for development, differentiation, survival, and homeostasis^16^. The mTOR kinase is a key regulator of autophagy. The class I PI3K/AKT signaling molecules link receptor tyrosine kinases (RTKs) to mTOR activation and repress autophagy in response to insulin-like and other growth factor signals^17^. In addition to mTOR, other regulatory molecules, such as 5′-AMP-activated proteinkinase (AMPK), BH3-only proteins, p53, death-associated protein kinases (DAPks), the inositol 1,4,5-trisphosphate receptor (IP3R), GTPases and calcium, can also regulate autophagy^18^. The role of autophagy in cancer and antitumor therapeutics has been extensively investigated during the last decade. Recent studies have shown that autophagy plays a role in tumor cell survival and cell death^19^,^20^,^21^.

In this study, we examined the effects of vandetanib on NSCLC cell line Calu-6 and the mechanisms underlying these effects. Our results showed that vandetanib inhibits cell migration and invasion. However, vandetanib also induces autophagy through reactive oxygen species (ROS) to antagonize the inhibitory effects on tumor cell growth. Inhibition of ROS or autophagy enhances the sensitivity of Calu-6 cells to vandetanib. Our results present new possibilities for the pharmacological targeting of NSCLC and introduce a novel role for vandetanib in treatment options.

## Results

### Vandetanib affects the cell morphology and the reorganization of the actin cytoskeleton and cell junctions in Calu-6 cells

**Figure 1:**
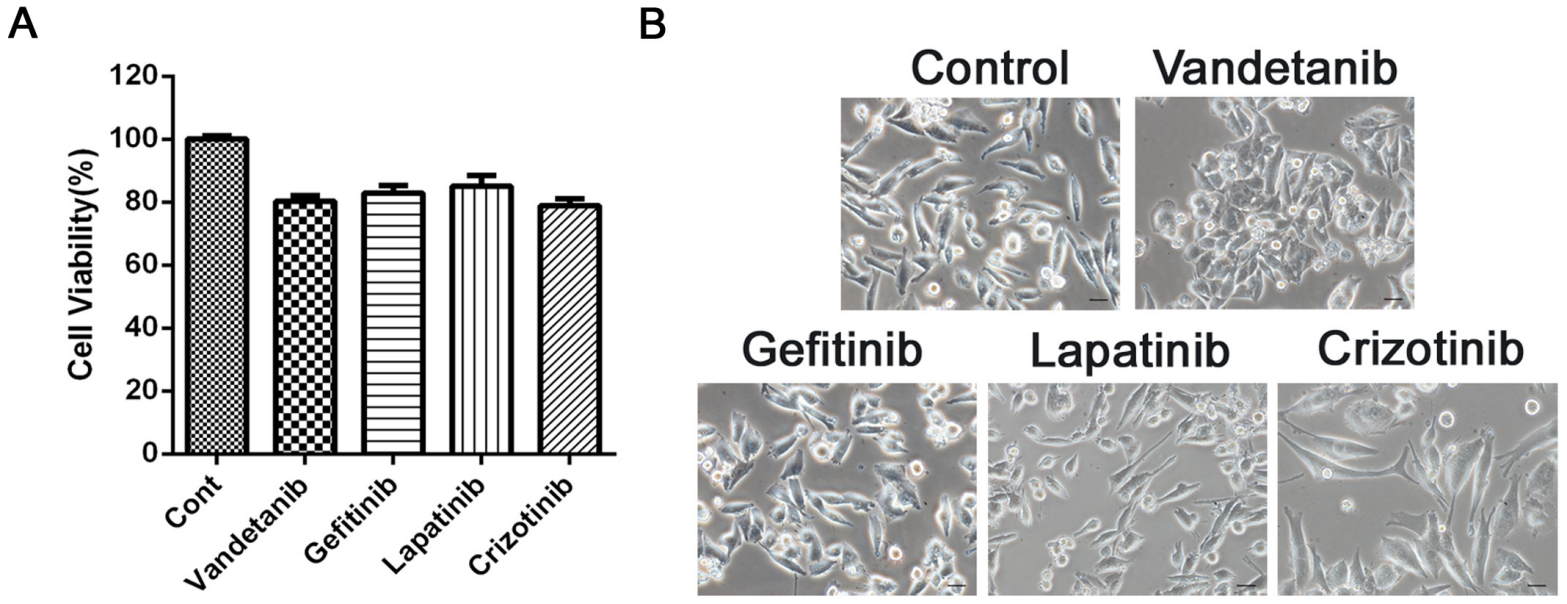
Vandetanib alters the mesenchymal morphology of Calu-6 cells. (A) Calu-6 cells were incubated for 24 h in the presence or absence of vandetanib (1 μM), gefitinib (1 μM), lapatinib (1 μM) or crizotinib (1 μM). Cell viability was measured using the CCK8 assay. (B) Calu-6 cells were treated as described above, and their morphology was examined with a light microscope. Scale bar: 50 μm.

**Figure 2:**
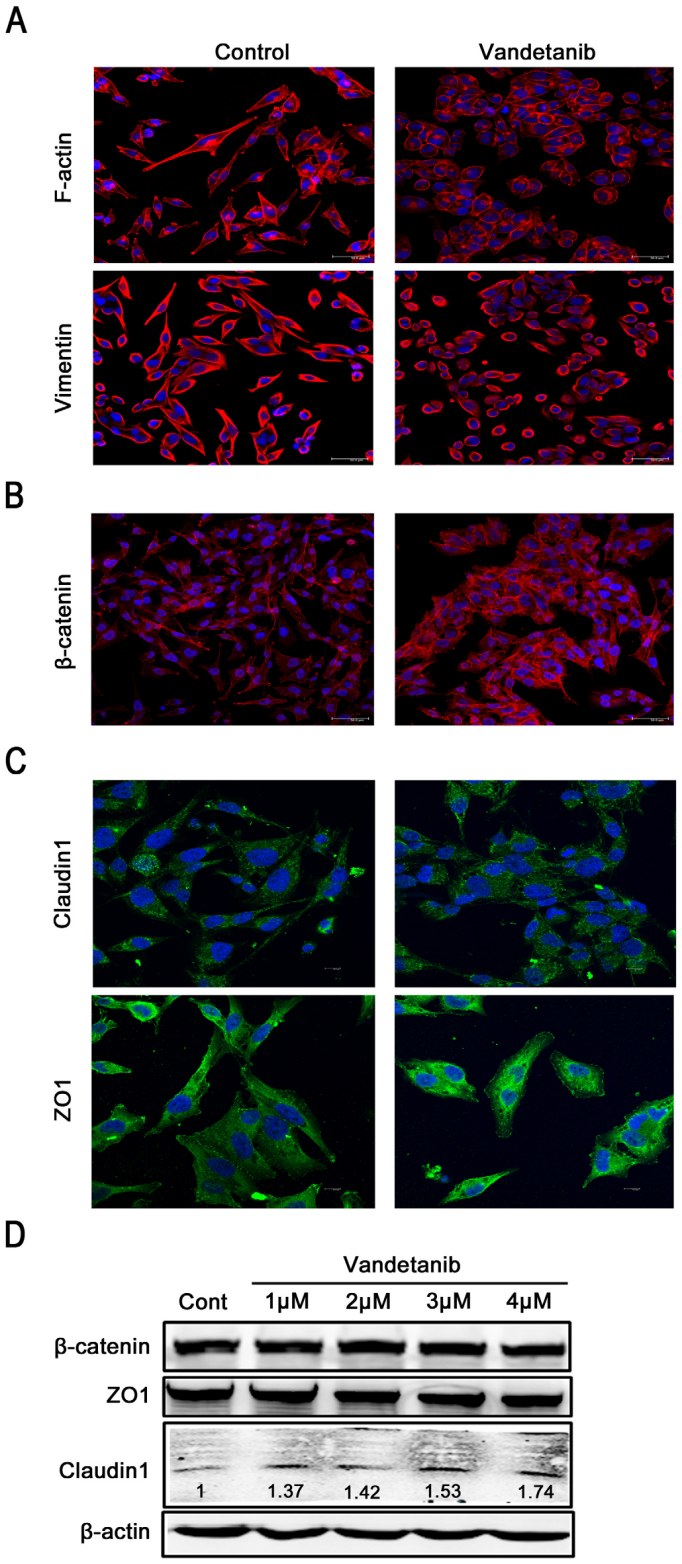
Vandetanib affects the actin cytoskeleton and cell junctions of Calu-6 cells. (A) Calu-6 cells were treated with or without 1 μM vandetanib for 24 h. F-actin and vimentin staining was performed as described in the Methods section and imaged using a confocal laser scanning microscope. Scale bar: 50 μm. (B) Calu-6 cells were treated as described above. Immunofluorescent staining of β-catenin was observed using a confocal microscope. Scale bar: 50 μm. (C) Calu-6 cells were treated as described above. Immunofluorescent staining of claudin1 and ZO1 was observed with a confocal microscope. Scale bar: 50 μm. (D) Cell lysates were prepared 24 h after the addition of vandetanib (1 μM, 2 μM, 3 μM and 4 μM) to Calu-6 cells. Untreated cells grown for 24 h were employed as a control. Western blot analysis was performed using specific antibodies against β-catenin, ZO1 and claudin1. β-actin was employed as the loading control. The protein levels of claudin1 were quantified relative to the loading control using Alphaview SA software. The presented blots were derived from multiple gels. The membranes were cut based on molecular weights and probed with the antibody of interest.

We chose TKIs including vandetanib, gefitinib, lapatinib, and crizotinib for the present study based on ongoing clinical trials of NSCLC in China, as the efficacy of these drugs is still uncertain. First, we examined the effects of these TKIs on cell death in the NSCLS cell line Calu-6, which expresses mutated KRas, but wild-type EGFR^22^,^23^. As seen in Figure 1A, none of these TKIs significantly inhibited the cell viability of Calu-6 cells. Only treatment with vandetanib resulted in a change from a mesenchymal-like morphology to an epithelial-like phenotype in Calu-6 cells (Figure 1B). This phenotype resembled mesenchymal-epithelial transition (MET), the reverse process of epithelial-mesenchymal transition (EMT). We therefore examined the mRNA levels of EMT markers including CDH1, CDH2, ZEB1, ZEB2, SNAIL1, SNAIL2 and BMI1 via q-PCR. Treatment of cells with vandetanib did not affect the mRNA expression of the EMT marker genes relative to control cells (Supplementary Figure S1), suggesting that vandetanib did not induce the MET observed in Calu-6 cells. The results of F-actin staining with rhodamine-phaloidin showed that vandetanib treatment resulted in a change from a spindle-like, fibroblastic morphology to a ‘cobble-stone'-like phenotype (Figure 2A *upper panel*), indicating a rearrangement of the cytoskeleton and the formation of cortical actin in the Calu-6 cells. Vimentin, a marker of intermediate filaments, exhibited a similar distribution to F-actin (Figure 2A *lower panel*). Treatment of Calu-6 cells with gefitinib, lapatinib, and crizotinib did not affect the rearrangement of F-actin and vimentin (Supplementary Figure S2 and Table S1). In addition, vandetanib affected cell junctions. Immunofluorescent staining for β-catenin, a marker of cell-to-cell adherens junctions, demonstrated that vandetanib-treated cells exhibited increased membrane accumulation of β-catenin compared with controls (Figure 2B). Similarly, vandetanib enhanced the membrane accumulation of the tight junction markers claudin1 and ZO1 (Figure 2C). We found that the protein levels of β-catenin and ZO1 were not upregulated in vandetanib-treated cells compared with the control cells (Figure 2D). However, vandetanib did cause concentration-dependent upregulation of protein expression levels of the tight junction marker claudin1 (Figure 2D), suggesting that vandetanib can remodel the actin cytoskeleton and cell junctions as well as altering the mesenchymal morphology of Calu-6 cells.

### Vandetanib inhibits cell migration and invasion

**Figure 3:**
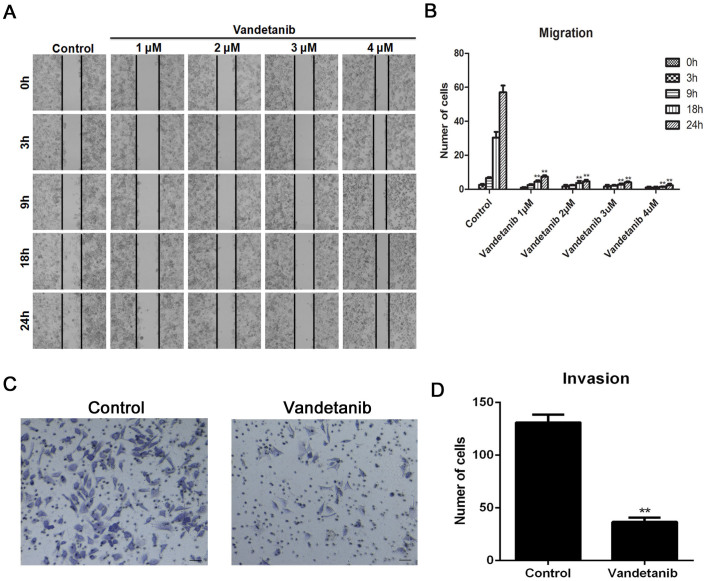
Vandetanib inhibits the cell migration and invasion of Calu-6 cells. (A) Calu-6 cells were treated with or without various concentrations of vandetanib, and their migration ability was subsequently determined using a wound-healing assay. Black solid lines denote the margins of the wound. Scale bar: 100 μm. (B) The number of cells that migrated to close the wounded area during 0 h, 3 h, 9 h, 18 h and 24 h were counted. The data are presented as the mean ± S.D. based on three independent experiments. **P < 0.01. (C) A 2-chamber assay was used to evaluate the invasion ability of Calu-6 cells. Scale bar: 100 μm. (D) The number of cells that travelled from the upper transwell chamber to the lower chamber was counted. The data are presented as the mean ± S.D. based on three independent experiments. **P < 0.01.

In contrast to epithelial cells, the mesenchymal morphology of cancer cells is a ‘transformed tumor cell phenotype', which endows cells with migratory and invasive properties. Thus, inducing a mesenchymal-to-epithelial-like morphology could inhibit tumor cell migration and invasion. To test this hypothesis, we performed a wound-healing assay and found that untreated Calu-6 cells converged from both sides of the wounded border from 3 h to 24 h (Figure 3A *first panel*). By contrast, few vandetanib-treated cells had migrated to close the wounded area at 24 h (Figure 3A *panels 2–5*), suggesting that vandetanib effectively inhibits cell migration (Figure 3B). Next, the effect of vandetanib on the invasive capacity of Calu-6 cells was evaluated using a transwell cell invasion assay. As shown in Figure 3C–D, vandetanib significantly decreased the invasiveness of Calu-6 cells.

### Vandetanib inhibits cell invasion through the Rho GTPases-JNK signaling pathway in Calu-6 cells

**Figure 4:**
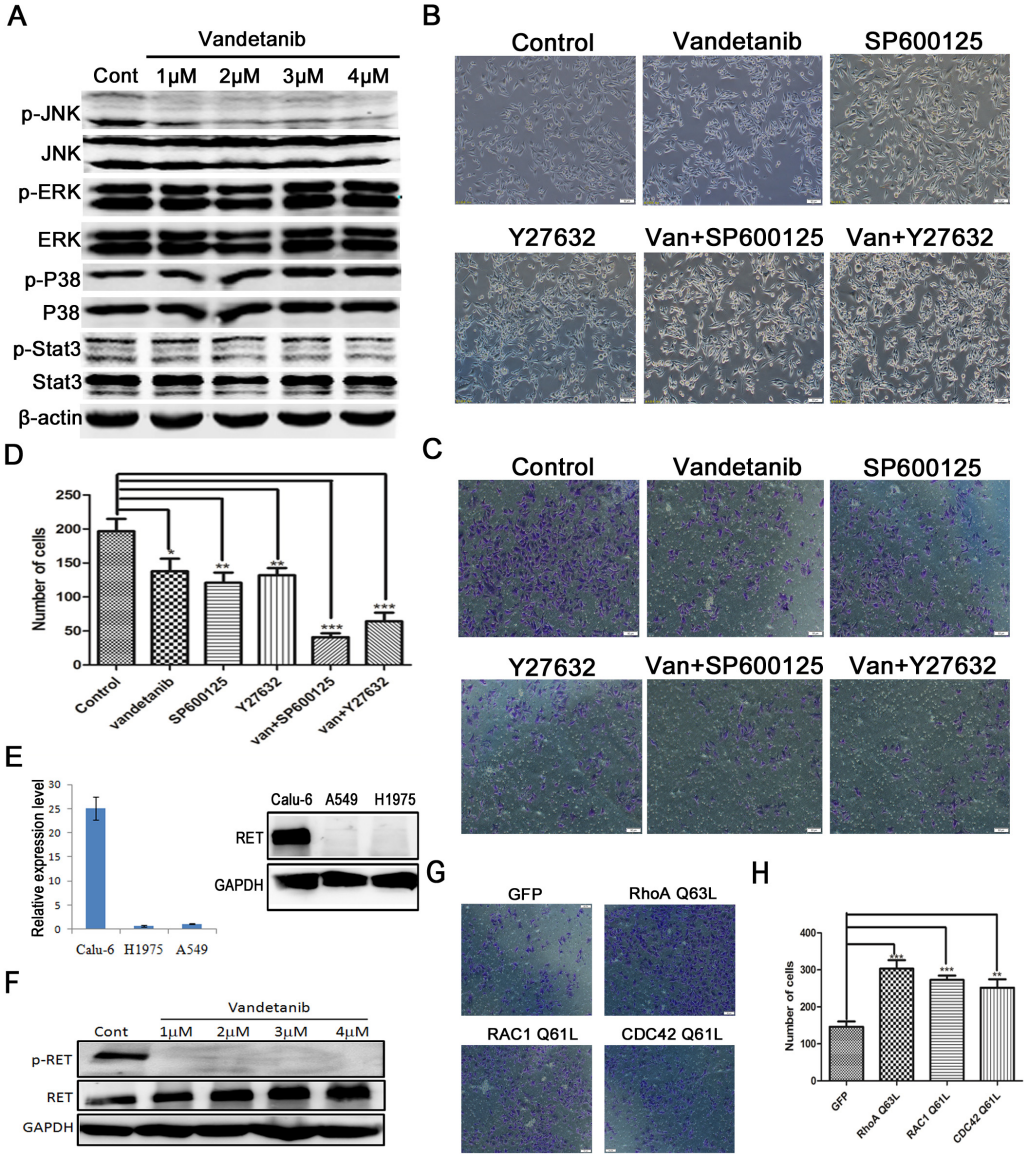
The Rho GTPase-JNK pathway is required for the inhibitory effects of vandetanib on Calu-6 cells invasion. (A) Calu-6 cells were treated with the various concentrations of vandetanib for 24 h and then analyzed by western blotting with antibodies against the phosphorylated or total forms of JNK, ERK, p38, and Stat3. β-actin was used as the loading control. (B) Calu-6 cells were incubated for 24 h in the presence or absence of vandetanib (1 or 2 μM), SP600125 (50 or 100 μM), and Y27632 (5 or 10 μM). The morphology of the Calu-6 cells was examined under a light microscope. Scale bar: 50 μm. (C) Calu-6 cells were treated as described above. Cell invasion was captured with a light microscope. Scale bar: 50 μm. (D) The number of invasive cells that travelled from the upper transwell chamber to the lower chamber was counted. The data are presented as the mean ± S.D. based on three independent experiments. *P < 0.05, **P < 0.01, ***P < 0.001. (E) Left: The mRNA expression levels of RET in different NSCLC cell lines were detected using q-PCR. Right: The protein expression levels of RET in different NSCLC cell lines were detected via western blotting. GAPDH was used as the loading control. (F) Calu-6 cells were treated as in Figure 4A and then analyzed by western blotting with antibodies against the phosphorylated or total form of RET. GAPDH was used as the loading control. (G) Calu-6 cells were transfected with plasmids carrying GFP, RhoA Q63L, RAC1 Q61L and CDC42 Q61L for 24 h, and the cells were then treated with 1 μM vandetanib for another 24 h. A 2-chamber assay was used to evaluate the invasive ability of the cells. The invasive cells were examined with a light microscope. Scale bar: 50 μm. (H) The number of invasive cells was counted. The data are presented as the mean ± S.D. based on three independent experiments. **P < 0.01, ***P < 0.001. Van: vandetanib. All of the presented western blots were derived from multiple gels. The membranes were cut based on molecular weights and probed with the antibody of interest.

We then investigated the molecular mechanism responsible for the inhibitory role of vandetanib. Intracellular mitogen-activated protein kinase (MAPK) pathways including P38, c-Jun NH2 terminal kinase (JNK) and extracellular signal-regulated kinase (ERK) pathways, are crucial for the regulation of cell migration and invasion^24^. MAPKs are also downstream signaling components of RTKs. Furthermore, some evidence indicates that the inhibitory effect of vandetanib on tumor cell proliferation and survival is mediated through inhibition of MEK/ERK and AKT signaling^25^. We therefore performed western blot analysis to determine whether vandetanib affects the expression levels of MAPKs. The results showed that vandetanib downregulated the protein levels of phosphorylated(p)-JNK in a concentration-dependent manner but did not alter the abundance of p-ERK and AKT (Figure 4A). To determine whether the inhibitory effect of vandetanib on p-JNK was unique to Calu-6 cells, we treated two other NSCLC cell lines, A549 and H1795, with vandetanib. We found that vandetanib treatment reduced the protein expression levels of both p-ERK and p-JNK in A549 and H1795 cells (Supplement Figure S3A) but did not alter the morphology of these two cell lines (Supplement Figure S3B), suggesting that vandetanib-induced inhibition of p-JNK is a general effect among NSCLC cells. Consistent with our results, an earlier study showed that the JNK signaling pathway can regulate the rearrangement of the actin cytoskeleton and control melanoma cell migration^26^. Next, we sought to determine whether inhibition of the JNK pathway was the downstream event required for the vandetanib-induced inhibition of Calu-6 cell migration. We therefore treated Calu-6 cells with SP600125, a JNK inhibitor that can also alter cellular morphology and inhibit cell invasion (Figure 4B–D). The combination of SP600125 and vandetanib treatment significantly enhanced the inhibitory effect on cell invasion (Figure 4B–D). We then explored how vandetanib suppresses JNK activity. As mentioned above, vandetanib is a multikinase inhibitor that targets EGFR, VEGFR and RET. Because gefitinib, an EGFR inhibitor, has no effect on the Calu-6 cell line, we speculated that the inhibitory role of vandetanib occurs through RET targeting. To test this hypothesis, we first examined the expression levels of RET in different NSCLC cell lines and found that RET was more highly expressed in Calu-6 cells (Figure 4E). This finding may partially explain why vandetanib affects the morphology of Calu-6 cells, but not other NSCLC cell lines, such as A549 and H1975. Next, we examined p-RET activity in Calu-6 cells following vandetanib exposure and found that 1 μM vandetanib completely inhibited the phosphorylation of RET at Y1062 (Figure 4F). Previous research has indicated that RET can directly interact with GTPase activating proteins (GAPs) to regulate the activity of small G proteins, such those of the Rho family^27^,^28^,^29^, which are known to act as upstream activators of the JNK pathway^30^,^31^. Similar to SP600125, the Rho-associated, coiled-coil containing protein kinase (ROCK) inhibitor Y27632 can also directly alter cellular morphology and inhibit cell invasion and has synergistic effects when combined with vandetanib (Figure 4B–D). To further confirm the critical role of Rho GTPase in the suppression of Calu-6 cell invasion following vandetanib treatment, we constructed plasmids carrying dominant positive mutants of the Rho family members RhoA Q63L, RAC1 Q61L and CDC42 Q61L and then transfected these plasmids into Calu-6 cells. After 24 h transfection, the cells were treated with vandetanib for another 24 h, and then used in cell invasion assay. The data showed that a constitutively active mutation in Rho GTPase, especially the RhoA Q63L mutation, significantly antagonize the inhibitory effects of vandetanib on Calu-6 cells invasion and JNK pathway activation (Figure 4G–H and Supplement Figure S4). Overall, these data suggest that vandetanib inhibits cell invasion through regulation of the RET-Rho- JNK signaling pathway in Calu-6 cells.

### Vandetanib induces autophagy in Calu-6 cells

**Figure 5:**
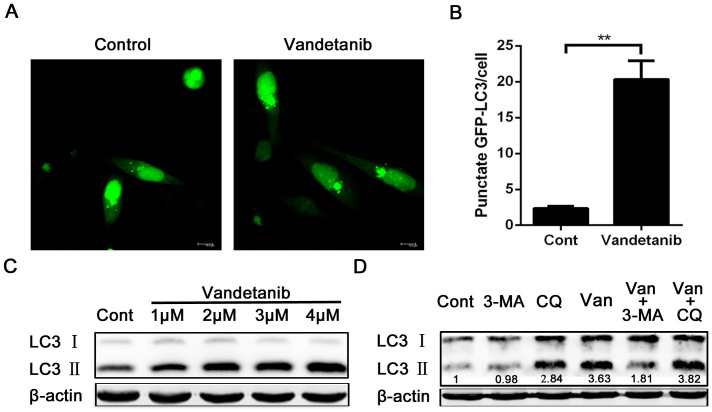
Autophagy was induced by vandetanib and inhibited by 3-MA and CQ in Calu-6 cells. (A) Calu-6 cells were transfected with the GFP-LC3 plasmid and then treated with 1 μM vandetanib for 24 h. GFP-LC3 puncta were examined via confocal microscopy. Scale bar: 50 μm. (B) The number of GFP-LC3 puncta in each Calu-6 cell was counted, and at least 100 cells were included for each group. The data are presented as the mean ± S.D. based on three independent experiments. **P < 0.01. (C) Calu-6 cells were treated with various concentrations of vandetanib for 24 h, and their whole-cell lysates were then subjected to western blotting with an anti-LC3 antibody. β-actin served as a loading control. (D) Calu-6 cells were treated with 1 μM vandetanib in the presence or absence of 5 mM 3-MA or 10 μM CQ for 24 h and then subjected to western blot analysis with an anti-LC3 antibody. The protein levels of LC3-II were quantified in relation to the loading control using Alphaview SA software. Van: vandetanib. The presented blots were derived from multiple gels. The membrane were cut based on the molecular weight and probed with the antibody of interest.

Despite evidence that vandetanib inhibits the migratory and invasive abilities of Calu-6 cells (and therefore has a potential antitumor effect), vandetanib use has resulted in paradoxical clinical outcomes when employed for the treatment of NSCLC. One interpretation of these findings is that there is a subset of NSCLC patients who will benefit from vandetanib and who can be identified through biomarker analysis^32^,^33^. In addition, we speculat that vandetanib may also play a protective role in NSCLC cells that accompanies its antitumor activity. Our previous work demonstrated that autophagy contributes to the chemoresistance of hepatocellular carcinoma cells^19^. Autophagy has been shown to be a mechanism of resistance to several chemotherapeutic agents, such as tyrosine kinase inhibitors and monoclonal antibodies targeting the EGFR and VEGFR signaling pathways in multiple solid tumor models including NSCLC. To determine whether vandetanib alters autophagy signaling, we examined vandetanib-treated Calu-6 cells that had been transfected with an expression vector encoding GFP-LC3. We found that GFP-LC3 was concentrated in autophagic vacuoles, as demonstrated by punctate fluorescence within the cells. Calu-6 cells that were either untreated or treated with vandetanib were transiently transfected with GFP-LC3 plasmids, and the results are shown in Figure 5A and 5B. A higher percentage of cells with punctate LC3 fluorescence staining were observed in the vandetanib-treated cells than in the control cells. We next analyzed the protein expression levels of LC3 via western blotting and found that vandetanib treatment leads to a significant increase in the expression of the autophagic form LC3-II in a concentration-dependent manner, indicating that autophagy is induced by vandetanib in Calu-6 cells (Figure 5C). Then, we investigated the effects of, 3-methyladenine (3-MA), which is an inhibitor of PI3K that inhibits autophagosome formation, and chloroquine(CQ), which can inactivate lysosomal hydrolases by inhibiting lysosomal acidification and thereby restrain autophagy flux. As shown in Figure 5D, 3-MA and CQ inhibited the autophagy response induced by vandetanib, demonstrating that autophagy is significantly activated in response to vandetanib treatment.

### Vandetanib activates autophagy via the enhancement of ROS levels in Calu-6 cells

**Figure 6:**
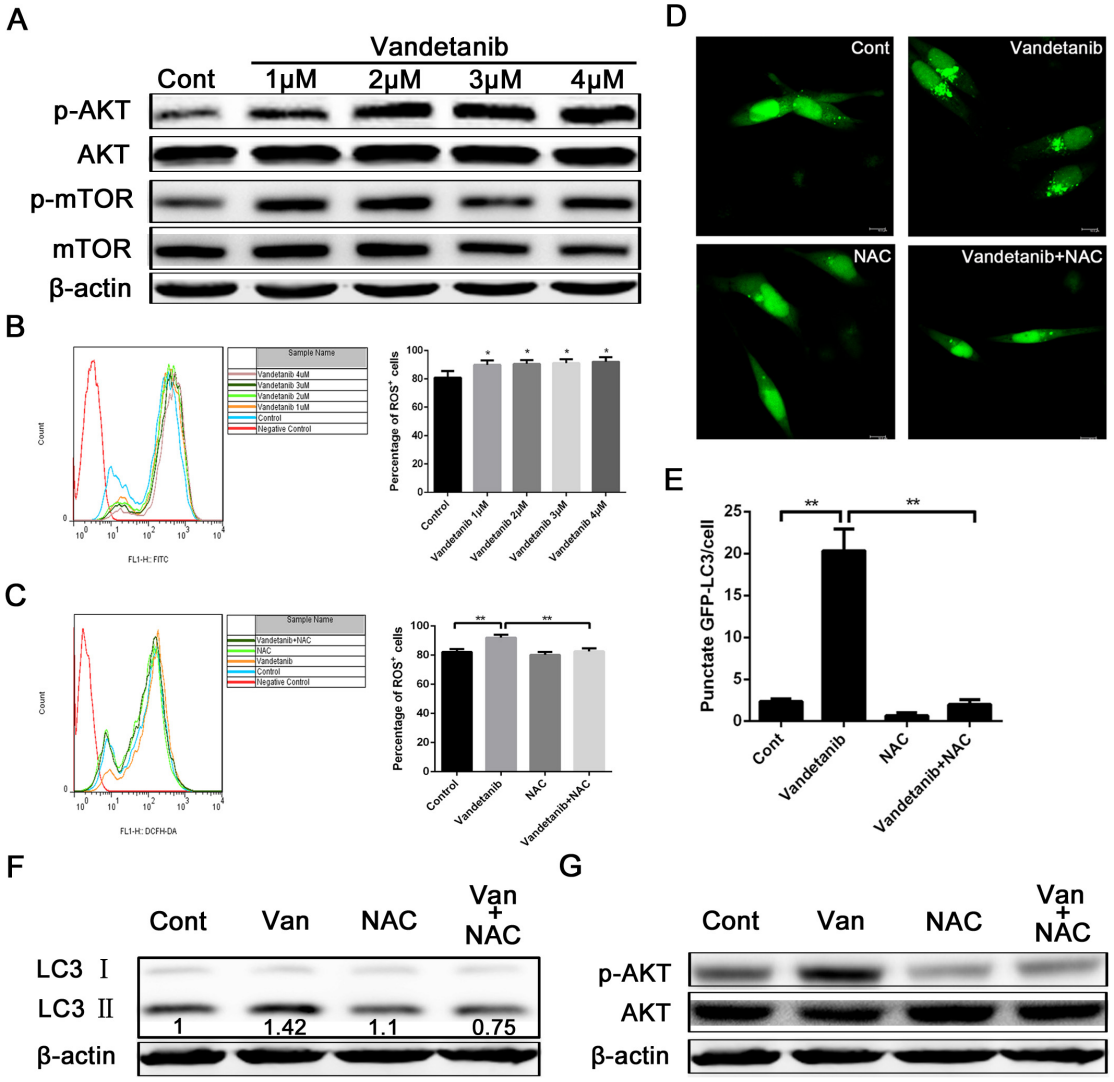
Autophagy induced by vandetanib is suppressed by inhibition of ROS in Calu-6 cells. (A) Cell lysates of Calu-6 cells were prepared following treatment with various concentrations of vandetanib for 24 h. Western blot analysis was performed to examin the expression of p-AKT and p-mTOR. β-actin was used as the loading control. (B) Calu-6 cells treated with or without various concentrations of vandetanib for 24 h were stained with 10 μM DCFH-DA and analyzed via FACS (left panel). The histogram represents the effect of vandetanib on ROS production (right panel). The data are presented as the mean ± S.D. based on three independent experiments. *P < 0.05. (C) Calu-6 cells were treated with 1 μM vandetanib in the presence or absence of NAC for 24 h and then stained with DCFH-DA and analyzed via FACS (left panel). The histogram shows the percentage of ROS^+^ cells induced by vandetanib (right panel). The data are presented as the mean ± S.D. based on three independent experiments. **P < 0.01. (D) Fluorescent microscopy images of GFP-LC3 in Calu-6 cells treated with 1 μM vandetanib in the presence or absence of NAC for 24 h. Scale bar: 50 μm. (E) The number of GFP-LC3 puncta in each Calu-6 cell was counted, and at least 100 cells were included for each group. The data are presented as the mean ± S.D. based on three independent experiments. **P < 0.01. (F) Calu-6 cells were treated with 1 μM vandetanib in the presence or absence of the ROS scavenger NAC (10 mM) and then subjected to western blot analysis to examine the expression of LC3. The protein levels of LC3-II were quantified in relation to the loading control β-actin using Alphaview SA software. (G) Cells were treated as described above and then subjected to western blotting to examine the expression of p-AKT and AKT. β-actin served as a loading control. The presented blots shown were derived from multiple gels. The membranes were cut based on molecular weights and probed with the antibody of interest.

The PI3K-AKT-mTOR signaling pathway is often activated by RTKs, and its inhibiton can induce autophgy^18^. As vandetanib directly inhibits RTKs activation, it may induce autophagy through suppression of the PI3K-AKT-mTOR pathway. To test this hypothesis, we examined the expression levels of p-AKT and p-mTOR via western blot analysis. Our results showed that vandetanib treatment increased the protein expression levels of p-AKT and p-mTOR in a concentration-dependent manner, which did not support our hypothesis and suggested that vandetanib-induced autophagy is mTOR-independent (Figure 6A). In addition to mTOR, ROS can also induce autophagy^34^. We therefore detected whether vandetanib could increase ROS levels in Calu-6 cells and found that vandetanib moderately enhanced the fluorescence intensity of ROS, but not in a dose-dependent manner (Figure 6B *left panel*). Furthermore, the percentage of ROS positive cells was increased in vandetanib-treated cells (Figure 6B *right panel*). We next evaluated whether an ROS inhibitior could suppress vandetanib-induced ROS production. N-acetyl cysteine (NAC), a general ROS scavenger, was added to vandetanib-treated cells for 24 h. As shown in Figure 6C, NAC effectively suppressed the ROS fluorescence intensity and the number of ROS-positive cells induced by vandetanib treatment. To determine whether enhanced ROS levels are required for vandetanib-induced autophagy, GFP-LC3 plasmids were transfected into Calu-6 cells for 24 h, and the cells were then incubated in the presence or absence of vandetanib and NAC for an additional 24 h. The cells were subsequently observed using a fluorescence microscope, and cells with GFP-LC3 puncta were counted. Our results demonstrated that NAC effectively inhibited the formation of GFP-LC3 puncta in vandetanib-treated cells (Figure 6D and 6E). In addition, examination of the protein expression levels of LC3 through western blotting produced results consistant with the fluorescent microscopy images (Figure 6F). Furthermore, NAC also decreased the expression levels of p-AKT activated by vandetanib (Figure 6G). These data indicated that vandetanib induces autophagy by increasing ROS levels in Calu-6 cells.

### Blockade of autophagy or ROS increases vandetanib-induced cell death in Calu-6 cells

**Figure 7:**
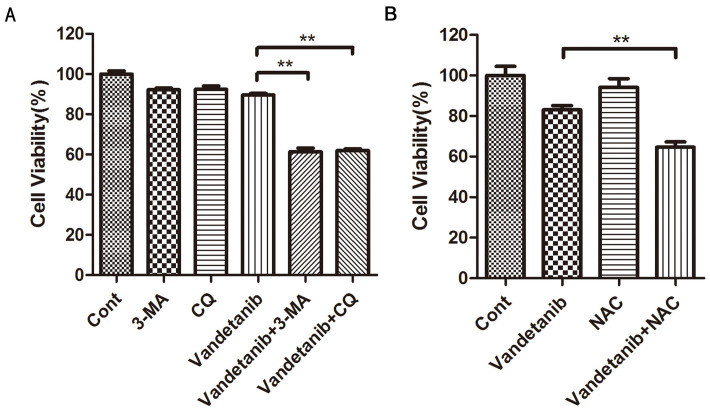
Inhibition of autophagy and ROS enhanced the chemosensitivity of vandetanib in Calu-6 cells. (A) and (B) Calu-6 cells were treated with 1 μM vandetanib in the presence or absence of 3-MA, CQ or NAC for 24 h. Cell viability was measured using the CCK8 assay. The data are presented as the mean ± S.D. based on three independent experiments. **P < 0.01.

Autophagy is regarded as a protective cell survival mechanism and vandetanib can induce autophagy by increasing ROS levels. Therefore, we speculated that inhibition of autophagy could increase vandetanib-induced cell death. As shown in Figure 7A, compared with single agent treatment, the combination of vandetanib and an autophagy inhibitor (3-MA or CQ) dramatically suppressed the cell viability of Calu-6 cells. Furthermore, NAC, an ROS scavenger, also increased vandetanib-induced cell death (Figure 7B). These results suggest that autophagy inhibition can enhance the sensitivity of Calu-6 cells to vandetanib.

## Discussion

**Figure 8:**
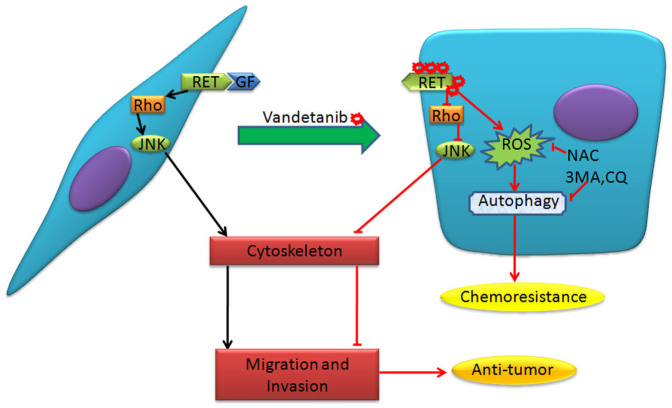
Molecular model of the effects of vandetanib on Calu-6 cells. Under normal culture conditions, growth factors (GF) interact with RTK, which then activates the Rho GTPase-JNK signaling pathway required for cell migration and invasion (left). Following vandetanib treatment, the fibroblastic morphology of Calu-6 cells is altered to a ‘cobble-stone'-like phenotype, and the capacity for cell migration and invasion is suppressed through inhibition of Rho GTPase-JNK signaling. Vandetanib also increases ROS levels, inducing autophagy and leading to chemoresistance. NAC or autophagy inhibitors enhance the chemosensitivity of cells to vandetanib (right).

In the present study, we demonstrated that vandetanib can alter Calu-6 cell morphology and regulate cell migration and invasion by directly inhibiting the Rho GTPase-JNK signaling pathway. However, vandetanib can also increase cellular ROS levels, inducing protective autophagy and contributing to chemoresistance (Figure 8).

The proto-oncogene RET encodes one of the receptor tyrosine kinases, which are cell-surface molecules that transduce signals for cell growth and differentiation. Targeted knockdown of RET leads to defects in neural crest development in a mouse model^35^. The major function of RET depends on the phosphorylation of its tyrosine (Y) residues, such as at Y1062^36^,^37^,^38^. The RET gene undergoes oncogenic activation through mutation or cytogenetic rearrangement in many tumors, but its role in cancer pathogenesis remains unknown. In this study, we found that the RET gene was highly expressed in the Calu-6 cell line. Although inhibition of RET activation by vandetanib treatment did not suppress cell growth, vandetanib did remodel the cellular cytoskeleton and enhance cell junction formation through impairment of the RET-Rho-JNK pathway. Tumor cells show peculiarities of invasion and metastasis when they gain the ability to detach from the primary tumor and enter into the surrounding tissue or lymphovascular channels, which is a critical step that depends upon disruption of the cellular cytoskeleton and cell junction^39^. Our results show that vandetanib can effectively inhibit Calu-6 cell invasion, indicating that RET may not be required for lung cancer formation but is necessary for tumor progression. Recently, numerous phase II and III trials have failed to show a meaningful difference in terms of outcomes when vandetanib is used for the treatment of NSCLC. Interestingly, the present study demonstrated that due to the expression of RET, the effectiveness of vandetanib was unique to the Calu-6 cell line, and vandetanib did not affect A549 and H1975 cells. These data imply that high expression of RET could be used as a biomarker of lung cancer patients who would benefit from vandetanib treatment. Coincidentally, another study showed that more than 50% of lung adenocarcinomas display a high incidence of copy number gains (3–4 copies) and amplification (≥5 copies) of the RET gene^40^. Therefore, selecting RET-positive lung adenocarcinoma patients for clinical trials employing vandetanib will be more likely to be successful^41^.

Originally, we speculated that vandetanib would suppress Calu-6 cell growth. However, our CCK8 assay demonstrated that vandetanib did not significantly inhibit cell growth. A previous study by our group suggested that autophagy can inhibit chemotherapy-induced cancer cell apoptosis^19^. Therefore, we examined the effect of vandetanib on autophagy in Calu-6 cells. Our results demonstrated that vandetanib induces autophagy in a concentration-dependent manner in Calu-6 cells, and inhibition of PI3K-AKT-mTOR activity is the core regulator of autophagy^18^. However, PI3K-AKT-mTOR activity was activated rather than suppressed in vandetanib-treated cells compared with controls. Therefore, vandetanib induces autophagy in a PI3K-AKT-mTOR-independent manner. PI3K-AKT signaling can be regulated by cell stressors such as ROS^42^ to induce autophagy^34^. In the present study, we demonstrated that vandetanib enhances ROS levels in Calu-6 cells. Treatment with NAC reduces PI3K-AKT activity and ROS-induced autophagy. Suppression of cellular autophagy or ROS enhances the sensitivity of Calu-6 cells to vandetanib, suggesting a promising therapeutic strategy for improving the chemotherapeutic options for NSCLC patients. However, some scientific questions still need to be answered, such as how vandetanib increases ROS levels, how vandetanib induces autophagy through ROS, and whether treatment with vandetanib in combination with NAC or autophagy inhibitors is effective in vivo. All of these issues deserve further investigation in the future studies.

## Methods

### Cell culture

The human NSCLC cell line Calu-6 was maintained in Dulbecco's modified Eagle's medium (DMEM) (GIBCO, Life Technologies, Cat.11995-065) supplemented with 10% fetal bovine serum (FBS) (GIBCO, Life Technologies, Cat.10099-141), 100 units/mL penicillin, and 100 mg/mL streptomycin (GIBCO, Life Technologies, Cat.15140-122). The cells were cultured under standard conditions at 37°C with 5% CO_2_ in a humidified incubator (Thermo Scientific). The following tyrosine kinase inhibitors were used: gefitinib (R&D Systems, Cat. 3000), lapatinib (BioVision, Cat.2138-25), vandetanib (BioVision, Cat.1751-25) and crizotinib (BioVision, Cat.1934-5). CQ (Sigma-Aldrich, Cat.C26628) and 3-MA (Sigma-Aldrich, Cat.M9281) were used at 10 μM and 5 mM, respectively. SP600125 (Selleck, Cat.S1460) and Y27632 (Selleck, Cat.S1049) were used at 50/100 μM and 5/10 μM.

### Cell Counting Kit-8

Cell viability assays were performed using the Cell Counting Kit-8 (CCK8) (DOJINDO, Japan). Cells (7 × 10^3^ cells/well) were first seeded into 96-well plates and incubated overnight. We then added the indicated agents to the medium and continued to culture the cells for 24 h. Next, 10 μL of the Cell Counting Kit-8 reagent was added to each well, and the cells were incubated for 1 h at 37°C. Finally, the spectrophotometric absorbance of each sample was measured using a microplate reader (Synergy HT, Bio-Tek) at 450 nm; based these readings, the percentage of surviving cells in each treated group was plotted. All of the experiments were carried out with six replicates.

### Immunofluorescence and confocal microscopy

Cells plated on coverslips were fixed with 4% paraformaldehyde for 20 min at ambient room temperature and permeabilized with 0.1% Triton X-100 for 10 min at room temperature. The cells were then blocked in PBS with 0.2% Tween 20 (PBST) containing 3% (w/v) bovine serum albumin (BSA) for 1 h at room temperature and incubated overnight with the primary antibodies against vimentin (abcam, Cat.2707-1), β-catenin (SANTA CRUZ, Cat.sc-7199) ZO1 (Proteintech, Cat.21773-1-AP) and claudin1 (Proteintech, Cat.10118-1-AP) at 4°C. We subsequently incubated the cells with Alexa Fluor 546 or 405 goat anti-rabbit IgG (Invitrogen, Cat.A-11035, Cat.A-31456) for 1 h at room temperature. F-actin filaments were stained using Texas Red-X phalloidin (1:1000, Invitrogen, Cat.T7471) for 45 min at room temperature. After the final washes with PBS, the coverslips were mounted onto microscope slides with ProLong Gold anti-fade reagent containing DAPI (Invitrogen, Cat.P-36931) for nuclear staining. The slides were examined, and images were captured using a confocal laser scanning microscope (Leica SP5). Each experiment was repeated at least three times.

### Western blot analysis

Cell lysates were extracted using a protein extraction reagent (Thermo Scientific, Cat.78501), supplemented with protease and phosphatase inhibitors (1:100, Roche Diagnostics). Equal amounts of protein were separated via SDS-PAGE and transferred to a nitrocellulose membrane (Whatman, Cat.10401396). The membranes were then blocked with blocking buffer (LI-COR, Inc., Cat.927-40000) for 1 h at room temperature and probed with the primary antibodies against β-catenin, claudin1 (Cell Signaling Technology, CST, Cat.4933), SAPK/JNK (CST, Cat.9252), phospho-SAPK/JNK (Tyr183/Ty185) (CST, Cat.4688), p44/42 MAPK (Erk1/2) (CST, Cat.4695), phospho-p44/42 MAPK (Erk1/2) (Tyr202/Tyr204) (CST, Cat.4370), p38 MAPK (CST, Cat.9212), phospho-p38 MAPK (Tyr180/Ty182) (CST, Cat.9211), Stat3 (CST, Cat.9132), phospho-Stat3 (Tyr705) (CST, Cat.9145), LC3B (CST, Cat.2775), AKT (CST, Cat.9272), phospho-AKT (Ser473) (CST, Cat.9271), mTOR (CST, Cat.2972), phospho-mTOR (Ser2448) (CST, Cat.5536), ZO1 (Proteintech,Cat.21773-1-AP), c-RET (SANTA CRUZ, Cat.SC-57431), Tyr1062-RET (SANTA CRUZ, Cat.sc-20252) and β-actin (HangZhou HuaAn Biotechnology Co., Ltd., China, Cat.R1207-1). After incubation with a fluorescently labeled secondary antibody (LI-COR, Cat.926-32211), the proteins were visualized using an Infrared Imaging System (ODYSSEY). β-actin was employed as a loading control, and the results are expressed relative to its levels. Each experiment was performed in triplicate.

### Wound-healing assay

Calu-6 cells were either treated with vandetanib (final concentration of 1 μM, 2 μM, 3 μM and 4 μM) or left untreated (controls). After incubation for 24 h, the cells were digested with 0.25% trypsin-EDTA (GIBCO, Life Technologies, Cat.25200-072), counted and plated at 1 × 10^5^ cells/well in 24-well dishes. The cells were then incubated overnight to yield confluent monolayers for wound testing. Wounds were made using a pipette tip, and the detached cells were removed by washing with PBS. Photographs were taken immediately (time zero) and at 3, 9, 18 or 24 h after wounding. We counted the number of cells that migrated to close the wounded area during this time period. These experiments were performed in triplicate.

### Transwell cell invasion assay

We used BD Biocoat Matrigel Invasion Chambers (BD Biosciences, Cat.354480) to conduct invasion experiments. This assay was carried out in 24-well transwell cell culture plates, with 8 μm pores. For inhibitor assays, 24 h after cells were treated with vandetanib, they received SP600125 or Y27632. For the plasmid assay, plasmids carrying GFP and Rho family members were first transfected for 24 h using lipofectamine 2000 (Invitrogene, Cat.11668-027) according to the manufacturer's protocol, after which the cells were treated with vandetanib for another 24 h. The cells were subsequently harvested and plated in complete medium in the upper transwell chamber at a concentration of 5 × 10^4^ cells/chamber in a volume of 0.5 mL. The lower chamber was loaded with 0.75 mL of culture medium containing 10% FBS. The 24-well plates were then incubated at 37°C under 5% CO_2_ for 24 h. Non-invading cells at the top of the transwell were scraped off with a cotton swab soaked in medium. Cells that had travelled through the pores (to the lower surface of the filters) were fixed with cold methanol for 30 min and then stained with a crystal violet solution (Beyotime, Cat. C0121) for 30 min. Invading cells were counted and imaged using a microscope at ×100 magnification. These experiments were carried out at least three times.

### Transient transfection and detection of autophagy

A GFP-tagged LC3 expression vector has recently been utilized to detect autophagy. Calu-6 cells were seeded (2 × 10^5^ cells/well) into 12-well plates overnight. GFP-LC3-expressing plasmids were then transiently transfected into the cells with Lipofectamine™ 2000 (Invitrogen, Life Technologies, Cat.11668-019) according to the manufacturer's instructions. At 24 h after transfection, the cells were treated with vandetanib and NAC (Beyotime, Cat.S0077). At the end of the treatment period, autophagy was examined by counting the percentage of cells with GFP-LC3 puncta using a confocal microscopy. We counted a minimum of 200 cells per sample, and each experiment was performed in triplicate.

### ROS measurement

To measure intracellular ROS, DCFH-DA (Beyotime, Cat.S0033) (used at a final concentration of 10 μM) was added to Calu-6 cells that had been treated with vandetanib or NAC for 24 h. After incubation at 37°C for 30 min, the medium was removed and the cells were washed three times with PBS. The cells were then analyzed using a FACS caliber flow cytometer (BD Biosciences). These experiments were carried out at least three times.

### Statistical analysis

Statistical analyses were performed using GraphPad Prism 5.0 software (GraphPad Software, San Diego, CA). The data are presented as the mean ± SD (standard deviation). Student's t-test was employed to detect differences in the mean values of the variables. P < 0.05 was considered as significant.

## Author Contributions

Y.Z., Y.L.Z. and H.B.Z. participated in the design and performance of this study. Y.Z. carried out cell culture. Y.Z. and Y.L.Z. performed western blot analysis. Y.Z. and N.C. carried out confocal microscope experiment. Y.Z. and X.J.C. performed cell migration and invasion assay. H.B.Z. carried out FACS analysis. Longmei Xu performed CCK8 assay. P.F.L. and X.M.K. supervised the work and provided financial support. The manuscript was drafted by Y.Z. and Y.L.Z., and reviewed by all authors. All authors approved the final version of the manuscript to be published.

## Supplementary Material

Supplementary Information Dataset 1
