# Supplementary material for: The multi-targeted tyrosine kinase inhibitor vandetanib plays a bifunctional role in non-small cell lung cancer cells
Source: Sci Rep. 2015 Feb 27;5:8629. doi: 10.1038/srep08629 (PMC4342569; doi:10.1038/srep08629)
Supplement: Supplementary Information — Dataset 1 [file srep08629-s1.doc]

**The multi-targeted tyrosine** [**kinase inhibitor**](http://en.wikipedia.org/wiki/Kinase_inhibitor) **vandetanib plays a bifunctional role in non-small cell lung cancer cells**

Yan Zhou^1,†^, Yuanliang Zhang^2,†^, Hanbing Zou^1,†^, Ning Cai^1^, Xiaojing Chen^1^, Longmei Xu^1^, Xianming Kong^1,3,*^ & Peifeng Liu^1,3,*^

1 Central Laboratory, Ren Ji Hospital, School of Medicine, Shanghai Jiao Tong University, Shanghai 200127, People’s Republic of China

2 Shanghai Institute of Hematology, Ruijin Hospital, School of Medicine, Shanghai Jiaotong University, Shanghai 200025, People’s Republic of China

3 State Key Laboratory of Oncogenes and Related Genes, Shanghai Cancer Institute, Renji Hospital, School of Medicine, Shanghai Jiao Tong University, Shanghai 200032, People’s Republic of China

***Corresponding author:**

1 Peifeng Liu, Central Laboratory, Ren Ji Hospital, School of Medicine, Shanghai Jiao Tong University, 1630 Dongfang Road, Shanghai 200127, People’s Republic of China. Tel.: +86-21-68383639. E-mail address:  [liupeifeng@yahoo.com](mailto:liupeifeng@yahoo.com)

2 Xianming Kong, Central Laboratory, Ren Ji Hospital, School of Medicine, Shanghai Jiao Tong University, 1630 Dongfang Road, Shanghai 200127, People’s Republic of China.Tel.: +86-21-68383639. E-mail address: [kongxianming@renji.com](mailto:kongxianming@renji.com)

^†^ These authors contributed equally to this work.

**
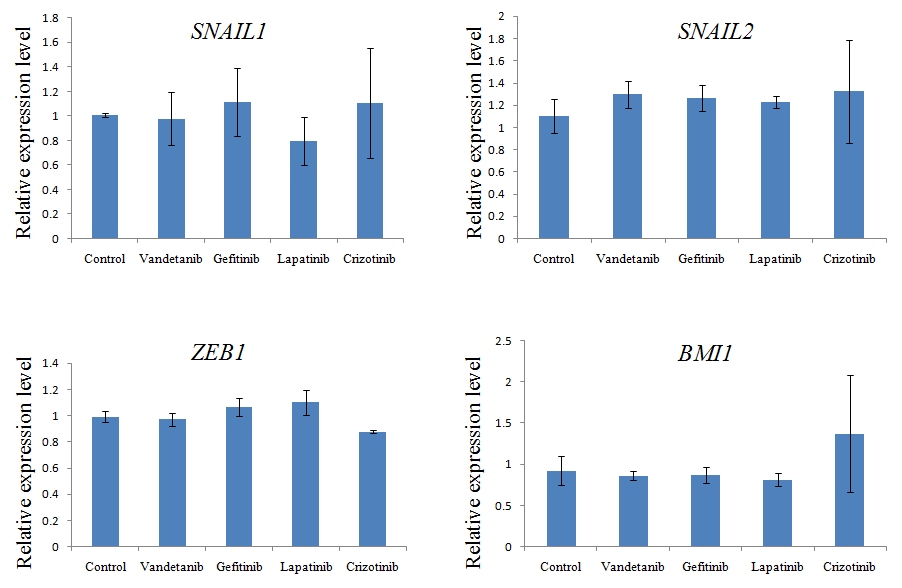
**

**Figure S1 Vandetanib was not required for MET of Calu-6 cells.** The mRNA expression levels of EMT related genes were examined in Calu-6 cells with or without vandetanib treatment via q-PCR assay. CDH1, CDH2 and ZEB2 were not expressed in Calu-6 cells.


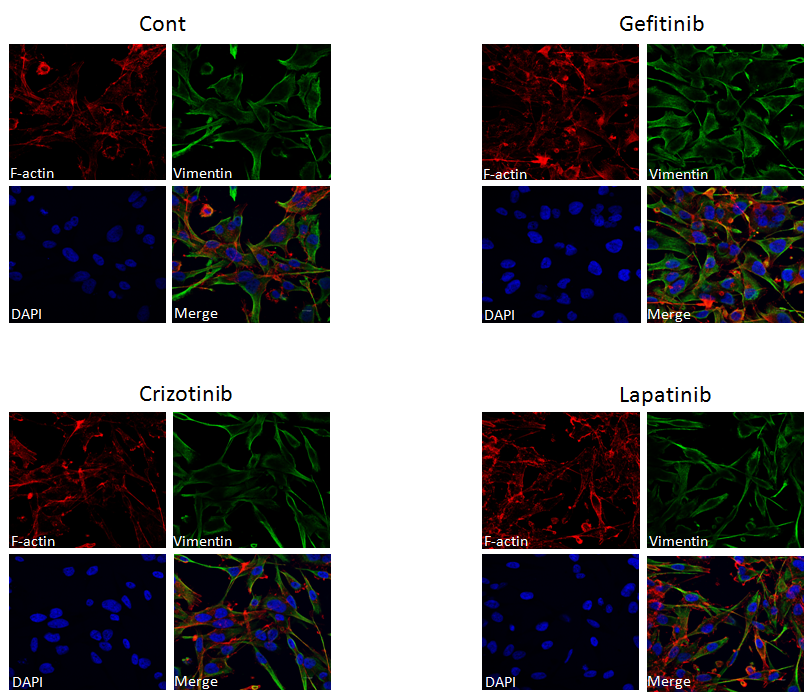


**Figure S2 Gefitinib, crizotinib and lapatinib did not affect the actin cytoskeleton of Calu-6 cells.** Calu-6 cells were treated with or without 1 μM gefitinib, crizotinib and lapatinib for 24 h. F-actin and vimentin were stained and imaged using a confocal laser scanning microscope. Scale bar: 10 μm.


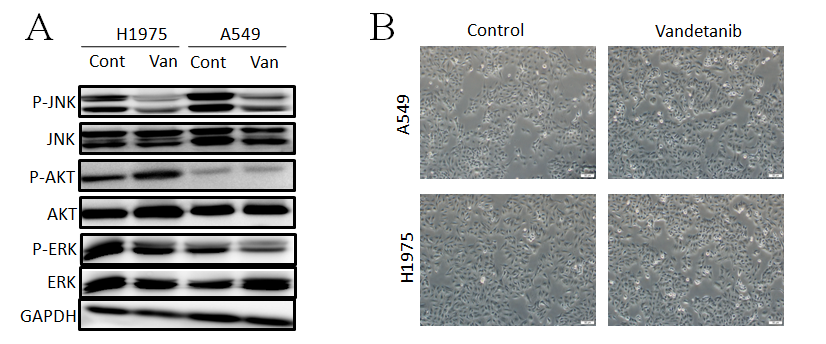


**Figure S3 The inhibition of JNK pathway by vandetanib was a general effect in NSCLC cell lines.** (A) A549 and H1975 cells were treated with or without 1 μM vandetanib for 24 h and then analyzed via western blotting with antibodies against the phosphorylated or total forms of JNK, ERK, and AKT. GAPDH was used as the loading control. The presented blots were derived from multiple gels. The membrane were cut based on molecular weights and probed with the antibody of interest. (B) The morphology of A549 and H1975 cells treated with or without 1 μM vandetanib for 24 h was examined by a light microscope. Scale bar: 50 μm.


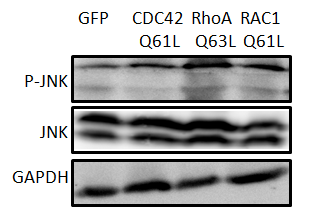


**Figure S4 JNK pathway was activated by dominant positive Rho GTPase mutation.** Calu-6 cells were transfected with plasmids carrying GFP, RhoA Q63L, RAC1 Q61L and CDC42 Q61L for 24 h, and then the cells were treated with 1μM vandetanib for another 24 h. The cells were subsequently harvested and analyzed by western blotting with antibodies against the phosphorylated or total form of JNK. GAPDH was used as the loading control. The presented blots were derived from multiple gels. The membranes were cut based on molecular weights and probed with the antibody of interest.

**Table S1: The number of fields captured and plates stained of immunofluorescent staining.**

**Agents NO. of fields captured NO. of plates stained**

| **F-actin** | **Control** | **4** | **3** |
| --- | --- | --- | --- |
|  | **Vandetanib** | **4** | **3** |
|  | **Gefitinib** | **4** | **2** |
|  | **Crizotinib** | **4** | **2** |
|  | **Lapatinib** | **4** | **2** |
| **Vimentin** | **Control** | **4** | **3** |
|  | **Vandetanib** | **4** | **3** |
|  | **Gefitinib** | **4** | **2** |
|  | **Crizotinib** | **4** | **2** |
|  | **Lapatinib** | **4** | **2** |
| **β-catenin** | **Control** | **4** | **3** |
|  | **Vandetanib** | **4** | **3** |
| **ZO1** | **Control** | **4** | **2** |
|  | **Vandetanib** | **4** | **2** |
| **Claudin1** | **Control** | **4** | **2** |
|  | **Vandetanib** | **4** | **3** |
